# A System for True and False Memory Prediction Based on 2D and 3D Educational Contents and EEG Brain Signals

**DOI:** 10.1155/2016/8491046

**Published:** 2015-12-24

**Authors:** Saeed Bamatraf, Muhammad Hussain, Hatim Aboalsamh, Emad-Ul-Haq Qazi, Amir Saeed Malik, Hafeez Ullah Amin, Hassan Mathkour, Ghulam Muhammad, Hafiz Muhammad Imran

**Affiliations:** ^1^Department of Computer Science, College of Computer and Information Sciences, King Saud University, Riyadh 11543, Saudi Arabia; ^2^Centre for Intelligent Signal and Imaging Research (CISIR), Department of Electrical & Electronics Engineering, Universiti Teknologi PETRONAS, Bandar Seri Iskandar, 32610 Tronoh, Perak, Malaysia; ^3^Department of Computer Engineering, College of Computer and Information Sciences, King Saud University, Riyadh 11543, Saudi Arabia; ^4^TeleNoc, Riyadh, Saudi Arabia

## Abstract

We studied the impact of 2D and 3D educational contents on learning and memory recall using electroencephalography (EEG) brain signals. For this purpose, we adopted a classification approach that predicts true and false memories in case of both short term memory (STM) and long term memory (LTM) and helps to decide whether there is a difference between the impact of 2D and 3D educational contents. In this approach, EEG brain signals are converted into topomaps and then discriminative features are extracted from them and finally support vector machine (SVM) which is employed to predict brain states. For data collection, half of sixty-eight healthy individuals watched the learning material in 2D format whereas the rest watched the same material in 3D format. After learning task, memory recall tasks were performed after 30 minutes (STM) and two months (LTM), and EEG signals were recorded. In case of STM, 97.5% prediction accuracy was achieved for 3D and 96.6% for 2D and, in case of LTM, it was 100% for both 2D and 3D. The statistical analysis of the results suggested that for learning and memory recall both 2D and 3D materials do not have much difference in case of STM and LTM.

## 1. Introduction

The multimedia technology has widely taken over the education system because it makes the concepts easy to understand. The multimedia principle by Mayer states that people learn more deeply from words and pictures than from words alone [1]. Currently, the dominant multimedia resources are mainly in 2D form. With recent advances in science and technology, 3D technology is becoming common and soon it will be available for educational purposes. As such, the educational contents can be presented to the students either in 2D or in 3D format. In 2D format, 3D objects are visualized by projecting them on 2D space. With the advent of 3D technology, nowadays, 3D objects can be visualized as 3D with the help of 3D devices. These advances in technology have now made us wonder whether the 3D material is more effective than the 2D content in terms of learning and memory retention and recall.

Electroencephalography (EEG) is an imaging technique that captures brain activation patterns detected as electrical activity on the scalp [2, 3]. EEG signals reflect the brain behavior during mental activities. Since the procedure of EEG is pain-free and noninvasive, it has been widely used in normal adults and children to study brain activation patterns associated with tasks like memory retention/recall, perception, attention, and emotions.

Some researchers studied the effects of 2D and stereoscopic 3D on tasks like viewer's experience of the movie content [4], spatial cognition [5–7], deepening the understanding of PC hardware [8], understanding and knowledge acquisition [9, 10], spatial visualization skills [11], and education learning processes [12]. These studies used subjective approach without using EEG brain signals. Also, no one explicitly studied the effects of 2D and 3D educational contents on learning and memory recall. Though the subjective approach based on the statistical analysis of the answers of questions can be used to assess the effects of 2D and 3D educational contents, EEG signals give direct insight into brain states and can lead to more accurate conclusions. Intuitively, the brain states are different while giving answers based on learned information and guess.

EEG technology has been employed by a number of researchers to study brain activations during different tasks such as cognitive tasks [13], playing video games on large screens [14], and playing video games on small and large displays [15]. Some researchers studied the EEG brain signals to locate brain regions and the components responsible for memory functions [16–19]. Also some studies have been done recently on attention, learning, and memory using EEG brain activations [20–23]. To the best of our knowledge, there do not exist any studies so far, which focused on studying the impact of 2D and 3D educational contents on learning and memory recall using direct brain behavior through EEG technology.

We studied the effects of 2D and 3D educational contents on memory retention and recall with the aid of brain images (topomaps) captured using EEG technology. For this purpose, we developed pattern recognition systems to predict true memory (remembered/recalled) and false memory (forgotten) using direct brain responses via topomaps for 2D and 3D educational contents and then used them for assessing their effects on learning and memory retention and recall. For developing the systems, the data was collected from sixty-eight healthy individuals. Half of them watched the learning material in 2D format whereas the second half watched the same material in 3D format. After learning task, memory retention and recall tasks were performed in the form of multiple choice questions (MCQs) related to the watched contents after 30 minutes (for STM) and two months (for LTM), and EEG signals were recorded.

The proposed pattern recognition systems comprise feature extraction and classification stages. Features are extracted from EEG signals corresponding to correct answer (true memory) and wrong answer (false memory) and SVM is used for prediction. For feature extraction, a simple and robust technique has been introduced that first of all creates topomaps from EEG signals and removes redundancy using city-block distance. One system was developed for 2D and 3D each to predict true and false memories, which can be used to predict the memory retention and recall abilities of individuals. Each system encodes the brain states and their performance has been analyzed to decide whether there is a difference between the impacts of 2D and 3D educational contents on memory retention and recall. In case of STM, the mean prediction accuracy is 96.5% for 2D and 97.5% for 3D, whereas it is 100% for both 3D and 2D for LTM. Further statistical analysis of the results revealed that there is no significant difference in using 3D and 2D materials as far as memory retention and recall is concerned. The initial results of this study were presented in [24]; this paper extends the study to LTM and gives thorough analysis of the proposed methods.

The main contributions of the paper are

- a pattern recognition system for predicting the state of memory, whether the memory is true or false, and
- assessment of the effects of 2D and 3D educational contents on memory retention and recall.

The remainder of the paper is organized as follows. The proposed methodology is elaborated in Section 2. The results are presented and discussed in Section 3 and Section 4 gives the statistical analysis.  Section 5 concludes the paper.

## 2. Methods and Materials

We developed one pattern recognition system for 2D and 3D each, for assessing the brain states corresponding to the true and false answers of MCQs, that is, true and false memories. Each system was trained with the labeled EEG brain signals that encode the brain behavior during true answer (true memory) and false answer (false memory). As both the systems have the same architecture and configuration and have been trained in the same way, the trained systems encode the brain behaviors corresponding to 2D and 3D contents and their prediction accuracies can be used to assess the effectiveness of 2D and 3D for learning and memory recall. To assess the effects of 2D and 3D, we developed the hypothesis that performance of the system associated with 3D is better than that of 2D. Precisely, the hypothesis is modeled as follows:

 
*Null Hypothesis H0*. Performance of the system associated with 3D is better than that of 2D.
 
*Alternate Hypothesis H1*. There is no difference in the performance of the systems associated with 2D and 3D.

Using hypothesis testing, we conclude whether the null hypothesis holds or not.

Our hypothesis about the difference between 2D and 3D groups is based on the following observations:

- For data collection, the subjects were selected in such a way that the age, IQ levels, and the background knowledge of the subjects in each group were same.
- Each group watched the same educational contents and responded to the questionnaires containing the same questions. The only difference was that of 2D and 3D formats.
- The brain states of each group are modeled as a classification system, which is based on the same feature extraction technique and the same classification method.
- Each system was implemented on the same platform using the same environment and was learned in the same way and was evaluated using the same procedure.
- There is complete similarity from data collection to learning and evaluating the systems. If there is any difference between 2D and 3D educational contents in respect of memory retention and recall, it should appear in the performance of the systems.

In the rest of this section, first, we give a general overview of the pattern recognition system. Then, we zoom into each component of the system. Feature extraction is an important component of the system, and discriminative features are needed to represent the brain behavior reliably corresponding to the answer of a question. We introduce a simple and robust method for feature extraction.

### 2.1. Proposed System

**Figure 1:**
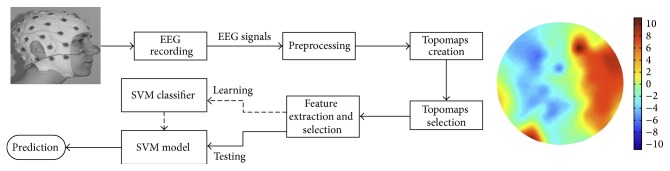
The proposed system for predicting true and false memories.

An overview of the proposed system is shown in Figure 1. First, subjects are selected to participate in the learning and memory recall tasks. EEG signals are recorded from the subjects during the tasks. These signals are preprocessed to remove the artifacts and noisy signals. Topomaps are then created from the clean signals, and after that the most representative topomaps are selected from the huge number of topomaps per sample (question). Different feature extraction approaches are used to extract the features from topomaps. Feature selection is needed to reduce the higher dimensionality of feature spaces and selects the most representative features, which subsequently are passed to the classifier to produce the classification model. Using the train classification model, we assess true and false memories and afterwards analyze the effects of 2D and 3D educational contents on learning and memory retention and recall.

In the following subsections, we give details of each step in the proposed system.

#### 2.1.1. Collection of Data

The details related to the data collection process are presented in this section.

*Selection of Participants.* A total number of volunteers who took part in the experiments were 68 and their ages were in the range of 18 to 30 years. The volunteers were having normal or close to normal eye sight and were not suffering from any form of neurological disorders, which could affect the results. Two groups were formed for 2D and 3D based on metrics of age and their knowledge.

*Learning Contents and Tasks Related to Experiments.* The same learning contents for both 2D and 3D related to the experiments in this study were acquired from Eureka 3D system [25]. The contents were of biology in nature and the volunteers had no existing knowledge in biological area. Learning and memory recall were two building blocks of the experiments performed. In learning phase, the participants watched 2D or 3D learning contents depending upon their group for the time spans of 8 to 10 minutes. In the phase of memory recall, two retention periods were employed, that is, one was for STM in which the retention period was of 30 minutes and the other was for LTM in which the retention period was of two months. In this process, twenty MCQs were inquired of the participants and each MCQ had four choices for answers. The same MCQs were asked to participants of both the groups, that is, 2D and 3D. While performing the recall experiment, EEG signals were recorded for study. The 41-inch TV screens were used for the display of learning contents and MCQs of the recall phase and those were placed at the distance of 1.5 m from the eyes of the participants. The participants had to answer the questions in the max. time of 30 seconds. In the case of right answers, “1” was assigned and in the opposite case “0” was assigned for the wrong ones.

**Figure 2:**
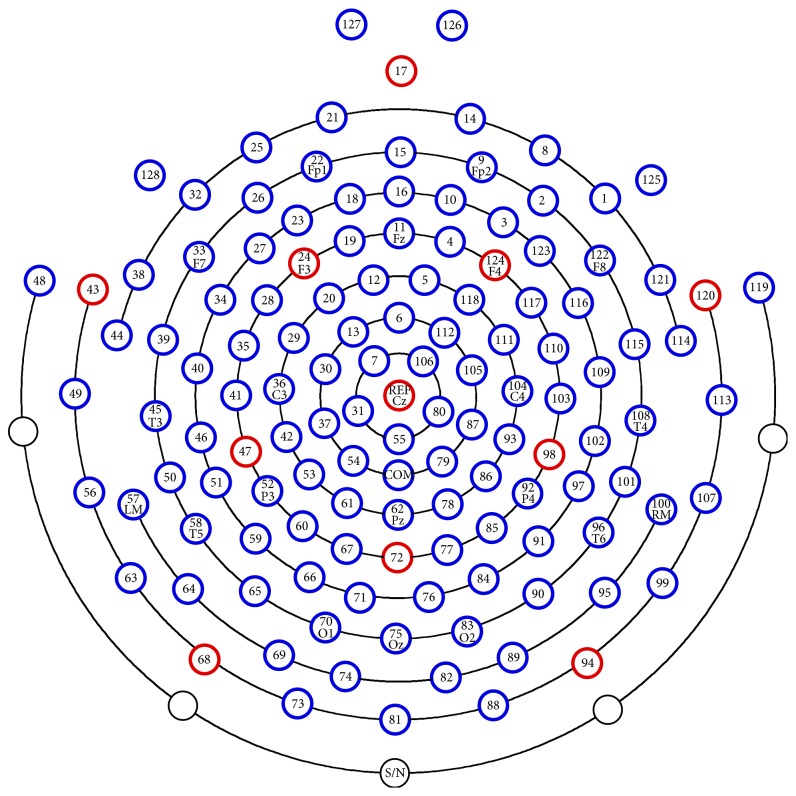
128-channel HydroCel Geodesic Net.

*Recording of EEG Signals.* The recording of EEG signals was done in the period of 30 seconds, which started when the participants were shown the questions and ended when they chose answers from the four choices for the answer. The starting and ending time points of the time period specified for answering the question were recorded in a file, which were used eventually for the extraction of the part of EEG signal related to the question. Sampling rates of 250 samples/sec and 128-channel HydroCel Geodesic Net (see Figure 2) were used for the recording of EEG data. The EEG signals are spatiotemporal data which are characterized by the temporal evolution and the adjacent spatial potential distributions. These signals are represented with *X*(*k*) ∈ *ℝ* ^*M*×*T*^ where *k* is the question identifier, *M* is the number of channels, and *T* is the number of sampled data points along temporal evolution. The selection of electrodes was performed based on their location on the scalp; that is, the outermost electrodes were omitted and in total 93 were chosen out of 128 electrodes for further analysis.

#### 2.1.2. Preprocessing

The noise in the form of artifacts, that is, eye blinking, and so forth is present in the recorded EEG data, which adversely affects the performance of feature extraction and eventually the prediction accuracy. It is utmost important to remove such noise for the overall performance of the system. In preprocessing phase, such noise is removed. The noise is present in various forms in the recorded EEG data, which includes the EEG activity that is not the result of response of stimuli; noise due to the variability in ERP components is a result of neural and cognitive activity variations; another common source of noise is the presence of bioelectric activities like movement of eyes, blinking, movement of muscles, and so forth; and the final source of noise is due to the electric equipment like display devices and so forth.

The artifacts in the raw EEG data were detected using a band pass filter (1–48 Hz) and then exported into MATLAB files (.mat) format using Net Station software of EGI. Ocular artifacts were removed by using Gratton and Coles method [26] and visual inspection.

#### 2.1.3. Creation of Topomaps

One possibility for feature extraction is EEG power spectral density, but it is computed in Fourier domain where time information is lost. We know that brain is a nonlinear dynamic system [27] and to keep track of the evolution of brain states over time is important. As such, we used EEG potential because it represents the changes of brain states over time. Recent studies have shown that time domain analysis plays an important role in understanding the EEG signals [28–31]. These studies have applied many time analysis methods on EEG recorded during performing various tasks (such as problem solving [29]) and brain states (e.g., emotional states [30] and epileptic seizure [32]). Hence, we have focused on the EEG potential rather than spectral analysis.

**Figure 3:**
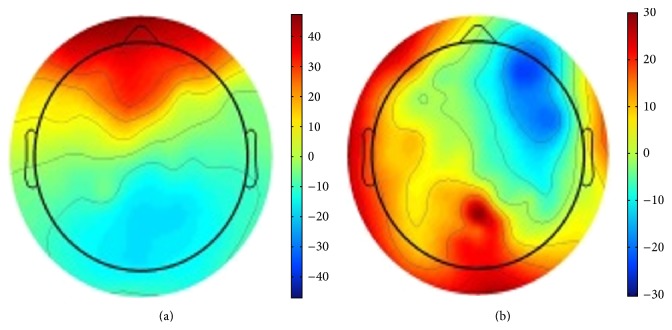
Two sample topomaps: (a) correct answer, (b) incorrect answer.

EEG signal at a particular time point *t* is a collection of the potentials at all electrode positions, that is, *x* ^*t*^ = (*x*_1_^*t*^, *x*_2_^*t*^, *x*_3_^*t*^,…, *x*_*M*_^*t*^), and is represented as a topomap (a digital image describing brain activation at *t*); two such topomaps are shown in Figure 3. EEG signals corresponding to the answer of each question are converted into topomaps. Features can be extracted from the topomaps using image processing and analysis techniques. The EEG signals are recorded from each subject while answering the questions and saved in a MATLAB raw file (.mat) and the time points at which the subject starts answering each question until finishing answering are saved in an event file. Using  .mat file together with the event file, we create the topomaps corresponding to the EEG recordings of each question. We used EEGLAB toolbox [33] for this step.

The quantity of topomaps depends upon the time which is taken by the participants for answering the questions. The number of topomaps corresponding to a question can be up to 7500 in total as the maximum time allocated for answering a question is 30 sec and sampling rate is 250.

#### 2.1.4. **Selection of Topomaps

**Figure 4:**
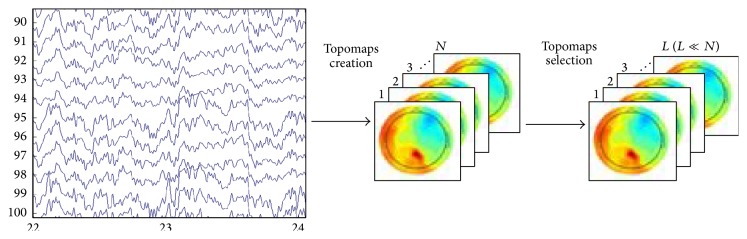
Selection of *L* topomaps with the highest dissimilarity.

Topomaps which occur consecutively in time contain redundant information, which do not aid in achieving good prediction accuracy. The more the discriminative information is, the more the prediction accuracy may be achieved. In order to have maximum discriminative information, the consecutive topomaps are analyzed using a distance metric and as a result the most discriminant ones are selected. A number of distance metrics have been proposed in the research community but city-block distance metric is well known for its simplicity and effectiveness and therefore we chose it for the selection of most discriminative topomaps. Using city-block distance metric, the distance between the pairs of topomaps is calculated and put in descending order, and finally the topomaps which are highly dissimilar are selected as shown in Figure 4.

#### 2.1.5. Feature Extraction

The next step after removing the redundant information is to extract discriminative features from the selected topomaps. First order statistics are used for the extraction of features from topomaps.

**Figure 5:**
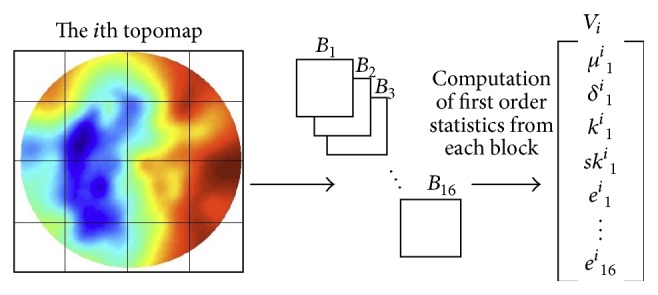
Features extraction from 4 × 4 blocks of topomaps.

In order to discriminate the structures in images, texture plays a major role. Texture information from the selected topomaps is extracted using first order statistics: mean, standard deviation, entropy, skewness, and kurtosis. First order statistics calculated from a topomap are global features and less discriminative because the localization information of the texture micropatterns is lost. For capturing localized texture information, each topomap is divided into a number of blocks, and then the statistical features are extracted from each block and merged into a vector that forms the representation of the topomap being processed. This process is shown in Figure 5. For our experiments, we tested different numbers of blocks but we found the acceptable number to be 8 × 8 and 16 × 16 blocks. We examined different combinations of five statistical features.

Similarly, we compute the feature vectors for each selected topomap and combine them into one feature vector that represents the brain state while answering a question. We can concatenate the vectors corresponding to all selected topomaps to form the feature vector, but in this case the dimension of the feature space will be excessively large causing curse of dimensionality problem. For instance, if 100 topomaps are selected, each topomap is divided into 16 × 16 blocks and 5 features are calculated from each block and then the dimension of the feature space will be 100 × 16 × 16 × 5 = 128000. We employ a different approach that reduces the dimension of the feature space significantly while keeping the most discriminative information and not depending on the number of selected topomaps. Using this approach, the dimension of feature space reduces to 4 × 16 × 16 × 5 = 5120 for the above case; details are given below.

After forming the vectors *V* ^1^, *V* ^2^,…, *V* ^*L*^ (each of dimension *s*) which contain features from the *L* most discriminant selected topomaps related to one question, the feature matrix (FM) of order (*s* × *L*) is built, where the vectors *V* ^1^, *V* ^2^,…, *V* ^*L*^ form FM columns. Equation (1a) shows FM for the case when only one feature (mean *μ*) is computed from each block. Now from each row of this matrix, the discriminative information is captured by computing four first order statistics (mean, standard deviation, skewness, and kurtosis); see (1b), and these are concatenated to form the feature vector as shown in (1a).

In (1a), FM of order *s* × *L* is the feature matrix for all topomaps corresponding to a question and *V* is the feature vector computed from matrix FM; in (1b), from the *i*th row of the matrix FM, four statistical features are extracted:

(1a) FMV1V2⋯VLμ11μ21⋯μL1μ12μ22⋯μL2μ13μ23⋯μL3⋮⋮⋯⋮⋮⋮⋯⋮⋮⋮⋯⋮μ1s−1μ2s−1⋯μLs−1μ1sμ2s⋯μLss×L⟶Vμ1δ1sk1k1⋮μsδssksks4s×1,

(1b) μ1i,μ2i,…,μLi⟶μi,δi,ski,kiT.

#### 2.1.6. Feature Selection

If a topomap is divided into 16 × 16 blocks and five statistical features are computed from each block, then each topomap will be represented by (5 × 16 × 16 = 1280) features and the total number of features will be 1280 × 4 = 5120 per question, which is high. High dimensional feature space has a number of disadvantages like high computational cost and affects the classification accuracy. It is utmost important to employ some feature selection methodology in order to reduce the high dimension of the feature space by reducing the redundancy and selecting the most discriminant features. In the proposed approach, ROC curve is used to select the most important features. The receiver operating characteristic (ROC) curve measures the class separability of a certain feature. It quantifies the overlap between the distributions of the feature in two classes and is computed as an area between two curves. This area is zero for complete overlap and is 0.5 for complete separation. A feature is discriminatory if the area for this feature is close to 0.5, for details see [34].

#### 2.1.7. Classification

Learning and memory recall process incorporates two classes of data, that is, correct and incorrect answers. For this problem, many learning models can be used, which include but are not limited to artificial neural networks, decision trees, and support vector machines (SVMs). Out of these, support vector machines are regarded as the-state-of-the-art classification models which can achieve outstanding accuracies. This is a linear classifier which achieves the maximum margin between the classes of data and can work for the very few training samples and at the same time works for classifying nonlinear data as well by taking the data to higher dimensional space using kernel trick. Various kinds of kernels can be used with SVM like radial basis function (RBF) and polynomial. As RBF kernel is known to give good results, we choose it for the classification purposes. The parameters of the kernel are learned using grid-search method and the well-known LIBSVM [35] library is used for the implementation of SVM. Note that SVM is useful for applications where the number of features is much larger than the number of samples [36–39].

## 3. Results and Discussion

In this section, the results of the proposed approach are presented and subsequently discussed. First, we describe the evaluation policy that we used to evaluate the systems. Then, we present an overview of the subjects and their answers to the questions in both STM and LTM sessions. After that, we present the result of the methods we used on STM and LTM. In the next section, we give the statistical analysis of the result of STM and LTM for 2D and 3D to see if there is a difference between them or not.

### 3.1. Evaluation Policy

In this section, the details about the dataset used for experiments are presented along with the evaluation methodology and the metrics used to measure the prediction accuracies are discussed.

For the experiments performed, the dataset is selected to make sure that the samples used for training and testing phases fairly represent the two classes. The selected set comprises 200 questions, out of which 100 are from correct class. The same procedure is used for data selection in both 2D and 3D learning contents for both the cases of STM and LTM.

Classification is performed between subjects so that the system is general and does not depend on a particular subject. Each system is learned and tested using the data across different subjects of the same group.

In order to estimate the prediction accuracy of the classifiers, we used 10-fold cross validation [40]. Cross validation is a renowned validation technique that is employed for such purpose. In 10-fold cross validation, data is randomly partitioned into 10 parts in which training is performed on nine parts and the left-over fold is used for testing purposes. This procedure is applied 10 times and in each turn different fold is used for testing purposes while the rest are used for training. At the end, to estimate the prediction accuracy of the system, mean and standard deviation of 10 iterations are calculated. This is the-state-of-the-art methodology for estimating the prediction accuracy of a classifier and ensures there is no overfitting because every time the system is trained with different data set and tested with different data set, not used in training.

In order to measure the performance of the systems, the well-known metrics of accuracy and the AUC were used. As we collected the data corresponding to 2D and 3D educational contents in the same way and also used the same classification models, which were trained and tested in the same way, the prediction performance of the classifiers is going to help in investigating the effectiveness of 2D or 3D learning contents for learning and memory recall.

### 3.2. Results for Long Term Memory (LTM)

We modeled two systems, one for 2D educational content and one for 3D case. We trained and tested each system using the method described in Section 3.1. In this case, each system involves three parameters, which include number of blocks, the extracted features from each block, and the number of selected topomaps.

Initially, we selected 20 topomaps and partitioned each topomap into 8 × 8 and 16 × 16 blocks. The results are shown in Tables 1 and 2. The best accuracy obtained in case of 8 × 8 is 90.5% for 3D with two features and 91.5% for 2D with five features, and it is 91.5% for 3D with two features and 95.5% for 2D with five features in case of 16 × 16 blocks. In case of 16 × 16 blocks, there is little increase in the accuracy but the overall trend is the same; that is, the best accuracy for 3D case is obtained with 2 features and that for 2D case is with features per block.

Next the number of selected topomaps was increased to 30 and features are extracted from 8 × 8 and 16 × 16 blocks. The results are shown in Tables 3 and 4. The best accuracy obtained in case of 8 × 8 is 96.5% for 3D with three features and 92.5% for 2D with three features, and it is 96% for 3D with two features and 97.5% for 2D with four features in case of 16 × 16 blocks. It indicates that, by increasing the number of selected topomaps, the accuracy increases.

In view of this trend, we increased the number of selected topomaps to 50, 100, 120, and 150. With 120 and 150 selected topomaps, we got the maximum accuracy. The results with 150 topomaps are shown in Tables 5 and 6. The best mean accuracy of 100% was achieved with 16 × 16 blocks and one feature (i.e., mean) per block for both 2D and 3D cases.

**Figure 6:**
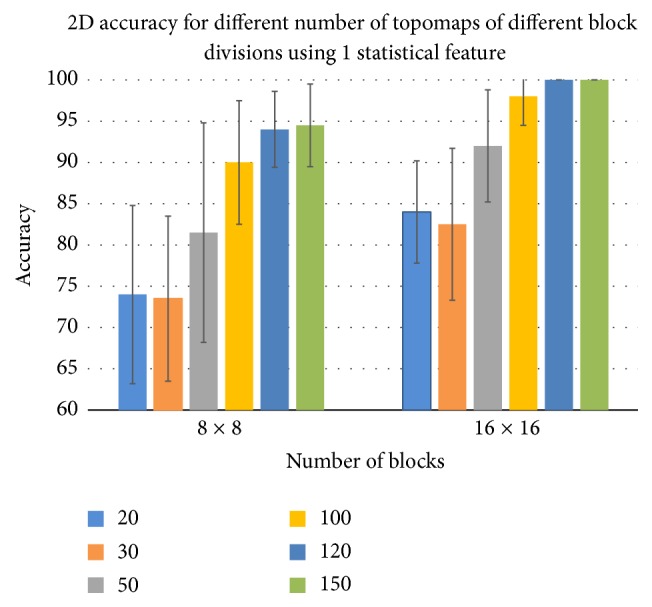
The best case accuracies in case of LTM for different selected topomaps for 2D educational material.

**Figure 7:**
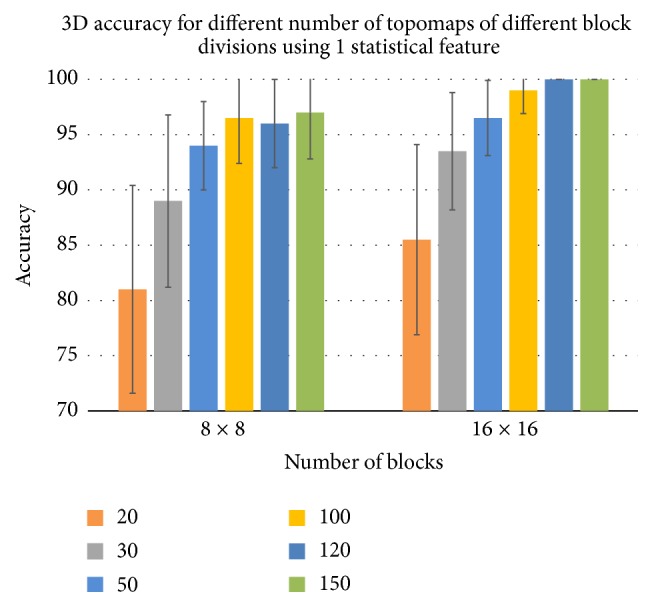
The best case accuracies in case of LTM for different selected topomaps for 3D educational material.

Generally, the more the number of topomaps and the number of blocks, the better the accuracy for both 2D and 3D cases as shown in Figures 6 and 7. The best average accuracy (100%) was achieved by 120 and 150 topomaps. The number of selected topomaps, which gives the best accuracy, is much lower than the total number of topomaps per question (less or equal 7500). It indicates that the discriminative information about the brain behavior for true or false memory is embedded in few topomaps. Further the best performing systems select 150 discriminative topomaps, divide each topomap into 16 × 16 blocks, and compute one feature (i.e., mean) from each block. The other performance metric, that is, AUC, also shows the similar performance for both 2D and 3D cases.

The reason that the average accuracy of 100 was reached in case of LTM is that, after two months of retention, subjects either still remember the answers or forgot them at all, that is, clear separation of correct and incorrect answers.

### 3.3. Results for Short Term Memory (STM)

The results for STM were presented in [24]; the details can be found there. We tried 20, 30, 50, 100, 120, and 150 topomaps with 8 × 8 and 16 × 16 blocks. We present here only the results of the best case, which were obtained using 150 topomaps. The results are shown in Tables 7 and 8. The best accuracy obtained in case of 8 × 8 is 97.5% for 3D with three features and 95.5% for 2D with two features, and it is 97.5% for 3D with two features and 96.5% for 2D with also two features in case of 16 × 16 blocks. In this case, the configuration of the best performing systems for the prediction of true and false memories is 150 most discriminative topomaps, 16 × 16 block division, and two features (mean and standard deviation) from each block.

**Figure 8:**
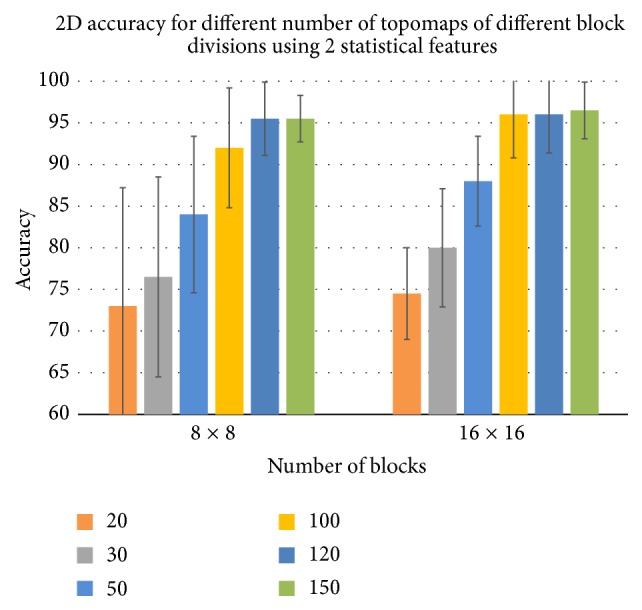
The best case accuracies in case of STM for different selected topomaps for 2D educational material.

**Figure 9:**
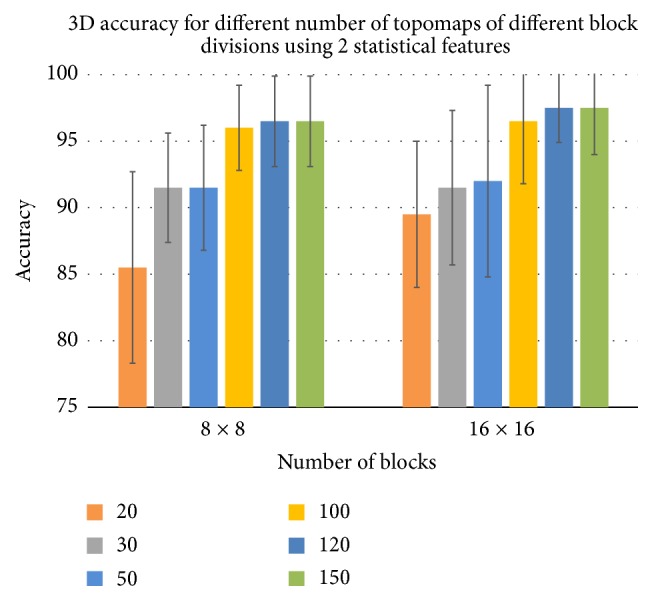
The best case accuracies in case of LTM for different selected topomaps for 3D educational material.

It was observed that prediction accuracy is directly proportional to the number of selected topomaps and reached to its peak for 150 topomaps. The primary factor for this behavior is that when higher numbers of topomaps are selected, we get more discriminative information. The results of the best case for all the selected topomaps with two different block divisions have been shown in a comprehensive way in Figures 8 and 9. Generally, the more the number of topomaps and the number of blocks, the more the accuracy of 2D and 3D.

### 3.4. STM + LTM

Now, we merged the 200 samples for STM with the 200 samples for LTM together and extract the statistical features. The average prediction accuracy is still excellent; we got an average accuracy of 97% for both 2D and 3D with the use of five features. This indicates that the proposed system is robust and is not affected by the size of the data.

## 4. Which Is Better 2D or 3D?

In order to come to any conclusion about the effectiveness of 2D or 3D educational contents for learning and memory retention/recall, we tested the hypothesis described in Section 3. For this purpose, we need enough samples of prediction accuracy values. To achieve this, we executed the systems five times with 10-fold cross validation using the best parameters and every time randomizing the datasets so that we can get average accuracy values for 50 samples having different combinations of true and false memories. After getting 50 accuracy values for the systems developed for 2D and 3D, we used SPSS for hypothesis testing.

Using SPSS software, an independent *t*-test was applied depending on the assumption that normality is acceptable. The results for 2D and 3D groups are approximately same; that is, in case of 2D, M is 96.6 and SD is 3.7, while in 3D case M is 97.2 and SD is 3.3. The value of *t* is −0.847 and *p* is 0.399, which is greater than 0.05. Therefore, the null hypothesis is rejected and it can be said that statistically 2D and 3D contents are same for learning and memory recall in the case of STM. Similar tests were performed in the case of LTM and same can also be said that 2D and 3D contents are statistically the same for learning and memory recall. In the case of 2D, M is 99.4 and SD is 1.6 and for 3D M is 99.3 and SD is 2.0, while the value of *p* is 0.787 and *t* is 0.271.

The observed results did not contradict with the results based on the answers to the questions of the 2D and 3D content for both STM and LTM. If we analyze the results, we find that, in case of STM, the difference between the correct answers is 23 for 2D and 3D group, which is equal to 3% of all answers, whereas this difference is only 3 (i.e., 0.468%) in case of LTM. This percentage is not significantly different if we take into account that some correct answers may be due to guess simply and not true memory. This supports our finding that 2D and 3D educational contents are approximately same for learning and memory retention and recall. These findings are similar to the previous subjective findings on the effectiveness of 2D and 3D contents on learning and knowledge acquisition [11, 14] and education learning processes [21] without using EEG technology.

## 5. Conclusion

We studied the effectiveness of 2D and 3D educational contents on learning and memory retention/recall using EEG brain signals. For this purpose, we proposed pattern recognition systems for predicting true and false memories and then used them for assessing the effects of 2D and 3D educational contents on learning and memory retention/recall by analyzing the brain behavior during learning and memory recall tasks using EEG signals.

For modeling pattern recognition systems, first, we collected the data. Sixty-eight healthy volunteers participated in data collection. Two groups corresponding to 2D and 3D were formed, which were based on the participants' ages and their knowledge. The data collection was done in two phases: learning and memory recall. During the learning phase, depending upon the group, the participants watched 2D or 3D contents for durations of 8 to 10 minutes. In order to check memory recall, two retention periods were used, that is, 30-minute period in case of STM and 2-month period in case of LTM. Each participant answered 20 MCQs which were same for both 2D and 3D groups and were about the learned contents. During the recall process, EEG signals were recorded.

For the pattern recognition systems, we introduced a simple and robust feature extraction technique that first converts the EEG signals (corresponding to the response of a question) into topomaps, selects the most discriminative topomaps, and then captures the localized texture information from the selected topomaps. For classification, we employed SVM with RBF kernel. The proposed systems can reliably predict true and false memories employing the direct brain activations in case of STM and LTM. We developed separate systems with the same architecture and configuration for 2D and 3D educational materials. As each system encodes the brain activations during memory retention/recall tasks, the performance of the system can be analyzed to assess the impact of 2D and 3D educational materials. Statistical analysis reveals that there is no significant difference between the effects of 2D and 3D educational materials on learning and memory retention/recall. Our findings are in accordance with the previous studies (without using direct brain activations) on the effects of 2D and 3D materials. However, our approach is based on the pattern recognition systems. As such, our research has the added advantage of proposing pattern recognition systems, which can be used to predict true and false memories and can be adopted to assess the learning levels of students. Though there is no difference in the effectiveness of 2D and 3D educational contents on learning and memory retention/recall from the performance perspective, different brain regions can be activated for 2D and 3D educational material. To investigate which brain regions are activated for 2D and 3D, educational materials are the subject for future work.

## Figures and Tables

**Table 1:** Results with 8 × 8 blocks and 20 selected topomaps.

# features	Accuracy		AUC	
	2D	3D	2D	3D
1	74 ± 10.8	81 ± 9.4	0.73 ± 0.13	0.84 ± 0.11
2	91.5 ± 5.8	**90.5 ± 6.4**	0.90 ± 0.09	0.89 ± 0.11
3	90 ± 4.7	89 ± 5.7	0.93 ± 0.07	0.90 ± 0.05
4	89.5 ± 3.7	89.5 ± 6	0.90 ± 0.08	0.91 ± 0.07
5	**91.5 ± 4.7**	88.5 ± 4.7	0.93 ± 0.06	0.93 ± 0.05

**Table 2:** Results with 16 × 16 blocks and 20 selected topomaps.

# features	Accuracy		AUC	
	2D	3D	2D	3D
1	84 ± 6.2	85.5 ± 8.6	0.82 ± 0.12	0.84 ± 0.14
2	92 ± 7.2	**91.5 ± 5.3**	0.93 ± 0.07	0.94 ± 0.07
3	95.5 ± 6	88 ± 7.2	0.96 ± 0.08	0.89 ± 0.08
4	95 ± 4.1	88.8 ± 8.2	0.96 ± 0.04	0.90 ± 0.06
5	**95.5 ± 4.4**	88.5 ± 7.1	0.96 ± 0.04	0.89 ± 0.08

**Table 3:** Results with 8 × 8 blocks and 30 selected topomaps.

# features	Accuracy		AUC	
	2D	3D	2D	3D
1	73.5 ± 10	89 ± 7.8	0.73 ± 0.15	0.87 ± 0.11
2	92 ± 4.2	96 ± 4.6	0.89 ± 0.07	0.97 ± 0.06
3	**92.5 ± 6.4**	**96.5 ± 5.8**	0.94 ± 0.06	0.96 ± 0.06
4	92 ± 5.4	96 ± 3.9	0.92 ± 0.08	0.97 ± 0.03
5	91 ± 5.2	95.5 ± 4.4	0.92 ± 0.06	0.96 ± 0.04

**Table 4:** Results with 16 × 16 blocks and 30 selected topomaps.

# features	Accuracy		AUC	
	2D	3D	2D	3D
1	82.5 ± 9.2	93.5 ± 5.3	0.80 ± 0.12	0.94 ± 0.05
2	95.5 ± 3.7	**96 ± 3.2**	0.96 ± 0.06	0.97 ± 0.04
3	97 ± 3.5	94.5 ± 5.5	0.96 ± 0.05	0.95 ± 0.07
4	**97.5 ± 4.3**	93.5 ± 5.8	0.97 ± 0.06	0.96 ± 0.05
5	97 ± 2.6	94 ± 2.1	0.97 ± 0.03	0.95 ± 0.03

**Table 5:** Results with 8 × 8 blocks and 150 selected topomaps.

# features	Accuracy		AUC	
	2D	3D	2D	3D
1	94.5 ± 5	97 ± 4.2	0.94 ± 0.07	0.97 ± 0.04
2	98.5 ± 2.4	**100**	0.99 ± 0.02	1
3	98.5 ± 2.4	99.5 ± 1.6	0.98 ± 0.04	0.99 ± 0.01
4	**99 ± 2.1**	99 ± 2.1	0.99 ± 0.04	0.99 ± 0.03
5	99 ± 2.1	99 ± 2.1	0.99 ± 0.02	1

**Table 6:** Results with 16 × 16 blocks and 150 selected topomaps.

# features	Accuracy		AUC	
	2D	3D	2D	3D
1	**100**	**100**	1	1
2	99.5 ± 1.6	100	0.99 ± 0.01	1
3	99.5 ± 1.6	100	1	1
4	99.5 ± 1.6	99.5 ± 1.6	1	1
5	99 ± 2.1	99.5 ± 1.6	0.99 ± 0.02	1

**Table 7:** Results of 8 × 8 blocks using 150 selected topomaps.

# features	Accuracy		AUC	
	2D	3D	2D	3D
1	91 ± 7	87.5 ± 6.8	0.93 ± 0.06	0.87 ± 0.10
2	**95.5 ± 2.8**	96.5 ± 3.4	0.95 ± 0.05	0.96 ± 0.07
3	93 ± 5.4	**97.5 ± 3.6**	0.92 ± 0.07	0.99 ± 0.01
4	93.5 ± 5.8	96.5 ± 4.7	0.92 ± 0.08	0.98 ± 0.04
5	93.5 ± 3.4	97 ± 2.6	0.94 ± 0.07	0.97 ± 0.04

**Table 8:** Results of 16 × 16 blocks using 150 selected topomaps.

# features	Accuracy		AUC	
	2D	3D	2D	3D
1	95.5 ± 4.4	96 ± 3.9	0.97 ± 0.05	0.97 ± 0.04
2	**96.5 ± 3.4**	**97.5 ± 3.5**	0.95 ± 0.06	0.98 ± 0.03
3	86 ± 7.4	92.5 ± 5.9	0.87 ± 0.09	0.93 ± 0.08
4	85.5 ± 6.4	92.5 ± 7.6	0.86 ± 0.06	0.93 ± 0.08
5	86.5 ± 6.7	92 ± 6.3	0.88 ± 0.07	0.93 ± 0.07
